# “*It’s Like Juggling, Constantly Trying to Keep All Balls in the Air*”: A Qualitative Study of the Support Needs of Working Caregivers Taking Care of Older Adults

**DOI:** 10.3390/ijerph18115701

**Published:** 2021-05-26

**Authors:** Eline E. Vos, Simone R. de Bruin, Allard J. van der Beek, Karin I. Proper

**Affiliations:** 1 Center for Nutrition, Prevention and Health Services, National Institute for Public Health and the Environment, Antonie van Leeuwenhoeklaan 9, 3721 MA Bilthoven, The Netherlands; eline.vos.02@rivm.nl; 2 Research Group Living Well with Dementia, Department of Health and Wellbeing, Windesheim University of Applied Sciences, Campus 2, P.O. Box 10090, 8000 GB Zwolle, The Netherlands; sr.de.bruin@windesheim.nl; 3 Department of Public and Occupational Health, Amsterdam Public Health Research Institute, Amsterdam UMC, Vrije Universiteit Amsterdam, 1081 BT Amsterdam, The Netherlands; a.vanderbeek@amsterdamumc.nl

**Keywords:** informal care, family care, working caregivers, older adults, elderly, support, needs, preferences, qualitative research, focus groups

## Abstract

Many informal caregivers of older adults combine their caregiving tasks with a paid job. Adequate support is important to enable them to combine paid work with caregiving, while maintaining their health and wellbeing. To date, however, knowledge about working caregivers’ support needs is fragmented. This study, therefore, aimed to obtain more insight into the support needs of working caregivers of older adults. We conducted six online semi-structured focus group interviews with in total 25 working caregivers of older adults living at home. Data were complemented with information from seven working caregivers participating in the study’s advisory board. Data were analyzed using inductive and deductive thematic analysis. Six themes related to working caregivers’ needs were identified: (1) Recognition of caregivers, including the challenges they face; (2) Attention for caregivers’ health, wellbeing and ability to cope; (3) Opportunities to share care responsibilities; (4) Help with finding and arranging care and support; (5) Understanding and support from the work environment; (6) Technological support tailored to the needs and capacities of caregivers and older adults. To address these needs, working caregivers suggested several options in multiple domains of life (i.e., work, home and social life, care environment, personal health and wellbeing). To successfully support them, a multi-faceted effort, involving actors from multiple settings, is needed.

## 1. Introduction

Worldwide, many countries are experiencing a large growth in the number and proportion of older persons in their populations [1]. This poses challenges for the organization of long-term health and social care services. To cope with these challenges, governments stimulate people to live in their own homes until old age [2,3]. This aligns with the preference of many older people, who prefer to live at home for as long as possible [2,4]. Since the prevalence of frailty, multimorbidity and disability increases with age, the number of older adults living at home with health and social care needs is growing [3,5]. Increased care dependency in the home setting, however, implies a heavy reliance on informal care [6]. In the EU, an estimated 60% of care is provided by informal caregivers [7]. Informal or family caregiving, in this paper further referred to as ‘caregiving,’ refers to voluntary care and support to spouses, family members, friends or acquaintances needing help because of health problems or other limitations [8]. This may involve assistance with activities of daily living, such as doing household chores and running errands, washing and dressing, providing transport, administration or providing companionship. Moreover, due to the fact that older people tend to live longer at home with more complex health problems, the role of caregivers also increasingly expands to medical tasks and coordination of care [9].

In the Netherlands, one in three people aged above 16 years provide some form of informal care to someone in their immediate social setting, which also includes occasional help [10]. Around 15% provide informal care for longer than three months and/or at least eight hours per week [11]. One in four caregivers have paid work for at least 12 h per week, and thus, also actively participate in the labor market [12]. Due to the rising retirement age and progress in female labor participation, in the Netherlands, the number of caregivers combining care with paid work is expected to further increase in the future [6]. Caregiving can result in positive outcomes for the caregiver in terms of feelings of enjoyment, fulfilment and an improved relationship with the care recipient [13,14,15,16]. However, combining paid work and caregiving may also be challenging due to the additional tasks and resulting cumulative load, potentially leading to negative outcomes in multiple domains of life, for instance, increased disruption or strain in family life, feelings of stress and depressive symptoms, social isolation and financial strain due to economic costs of caregiving [17,18,19,20]. In the occupational domain, care provision has been associated with reduced working hours, increased sickness absence, other forms of absenteeism and even leaving employment altogether [8,19,21,22]. In the Netherlands, about one in four working caregivers report problems in combining paid work and informal care tasks, and one in five experience severe strain [23].

Adequate support for working caregivers is necessary to ensure their physical and psychosocial wellbeing, their labor force participation and, in turn, the central and important contribution they make to the sustainability of the long-term care system. Support options for working caregivers may vary between different countries [24]. In the Netherlands, several forms of support for caregivers are available, including psychosocial and educational support (e.g., training/education, counseling and respite care), support to combine paid work and informal care (e.g., care leave and flexible work arrangements) and financial support (e.g., tax benefits or other types of benefits) [25]. In recent years, there has also been an upsurge in the development of technological support measures for caregivers of older adults, such as apps for digital health monitoring, *domotica* (e.g., in-house alarms and sensors) and *robotica* (e.g., healthcare robots) [26,27].

While several forms of support for working caregivers of older adults are already available and caregivers emphasize that they have a need for support [28], in general, the uptake of existing support measures in practice is limited [28,29,30,31]. Caregivers are often poorly informed about possible support possibilities and tend to turn to support services at a late stage, when stress levels are already significant, making it more difficult to relieve the caregiver’s burden [29]. Moreover, the actual effects of caregiver support have also been found to be limited, which could be explained in part by the fact that interventions do not always take into account caregivers’ needs [32]. Literature regarding the effectiveness of support interventions differs considerably in terms of the focus, type, level and setting of the intervention or service that is examined, often focusing on a specific caregiving group, care demand, caregiver outcome or aspect of the caregiving process [21,30,33,34]. This fragmented knowledge and narrow focus makes it challenging to find a clear way forward in how to improve support for working caregivers in practice. Therefore, more insight is needed into working caregivers’ support needs across multiple domains of life, the challenges they encounter and their experiences with combining work, home life and care. Thus, this study aims to explore the support needs of various working caregivers who care for a care-dependent older adult living at home, based on their experiences and encountered challenges.

## 2. Materials and Methods

### 2.1. Study Design

This qualitative study was performed between June and October, 2020 in the Netherlands. We conducted six online synchronous focus group discussions (FGDs) [35] with working informal caregivers of older adults. This included one pilot session with five caregivers, in which we tested the duration of the discussion and the online experience of the participants. For the online discussions, we used the video conferencing application Cisco Webex Meetings (Cisco Webex, Milpitas, CA, USA, version 40.4.7). This method was chosen for safety reasons and because of travel restrictions during the data collection period due to the global outbreak of COVID-19. Online focus groups are seen as an efficient and convenient way to collect rich qualitative data through interactive group discussions, particularly with participants over a large geographical spread [36,37]. Furthermore, for triangulation purposes, we acquired data from additional sources in this study, including: (1) input from an advisory board, consisting of seven working caregivers (see Section 2.2), and (2) e-mail correspondence with two caregivers who were unable to join an online FGD, but voluntarily wanted to share some of their experiences and support needs via this way.

### 2.2. Recruitment of Participants

At the start of the project, we installed an advisory board of 10 working caregivers of older adults. During the course of the study, three of these caregivers dropped out due to lack of time, leaving seven working caregivers in our advisory board. The purpose of the advisory board was to contribute from start to end to the design and research questions of this qualitative study, as well as the dissemination of the results, from their own experiences as working caregivers. We recruited these participants by posting an announcement in two Facebook groups for caregivers. For the pilot session FGD, we approached working caregivers from the personal network of one of the researchers and snowball sampling. For the five remaining FGDs, we first approached caregivers who had signed up for the advisory board but could not be included due to a higher number of volunteers than needed. In addition, we recruited participants via caregiver social media groups and through newsletters of caregiver and elderly organizations, as well as local support centers for caregivers in different municipalities throughout the Netherlands. Because only a limited number of male caregivers responded, additional efforts were made to recruit extra male participants by approaching male caregivers who had expressed interest in attending an earlier meeting for caregivers related to this study, which had to be canceled due to the global outbreak of COVID-19. We also requested research partners to aid in the recruitment of male caregivers, and asked one already participating male caregiver to ask around in their network. These efforts only yielded one extra male participant.

Participants were eligible to participate in the advisory board or FGDs if they: (1) were aged 18 years or older; (2) provided care or had provided care in the previous five years to an older adult of 65 years or over, living at home (no minimum amount of caregiving required); (3) worked at least 20 h per week during the time of caregiving; and, for the FGDs, (4) had an electronic device with internet access for video conferencing. This meant that we could also include caregivers who had stopped working or had reduced their working hours due to the combination of work and care, and ask them about their support needs. The cutoff point of 20 h was chosen because we hypothesized that caregivers who work more than two days per week might have more support needs in combining paid work and care than those who work less. For the working caregivers in the pilot group no minimum number of working hours was required.

The Center for Clinical Expertise of the Dutch National Institute of Public Health and the Environment classified the study as exempt from ethical review as it did not meet the criteria of the Medical Research Involving Human Subjects Acts. The center approved the study protocol (study number VPZ-428). The study was performed in accordance with guidelines of good clinical practice and ethical principles as stated in the Declaration of Helsinki. Written informed consent was obtained from all participants.

### 2.3. Instruments

We used a semi-structured interview guide for the FGDs covering five main questions (Table 1). The questions were piloted in the advisory board set up for this study. Based on their suggestions, several questions were altered or rephrased, after which we shared these again with the advisory board members for final review. The five interview questions covered caregivers’ experiences and support needs as a working caregiver. For the question on the support needs of working caregivers (question 2, Table 1), we also employed an individual brainstorming task, where the participants first wrote down all of their support needs before sharing them with the group, one by one. The purpose of this task was to aid the participants in generating their support needs without influence from others and give them equal opportunity to share their ideas. Furthermore, we hypothesized that varying the discussion technique would make the online discussion more interactive and less intensive for participants. Caregivers willing to participate were asked to complete an online questionnaire on their socioeconomic characteristics, employment status and care situation.

### 2.4. Data Collection

The time of day of the FGDs was set in accordance with the participants’ schedules. In the pilot session, the participants were additionally asked how they had experienced the online session. No improvements were needed according to the pilot participants. The FGDs were conducted by two researchers: an experienced moderator (E.V.) and an observer (student researcher) who aided with the brainstorm activity and took notes. The sessions lasted approximately 1.5 h. Participants were asked to log in five to ten minutes before the starting time of the meeting, to check the video and audio connection of each participant. Additional data sources collected for triangulation were e-mail correspondence with two caregivers who could not participate in an FGD, as well as meeting notes and transcripts of two group sessions with the advisory board and their written feedback to questions of the research team.

### 2.5. Data Analysis

All focus group interviews were audio and video recorded using the built-in recording function of the Webex Meetings video conferencing tool. Participants reaffirmed consent verbally prior to the recording of the interviews. Afterward, the audio track was separated from the video track and used to transcribe the discussions verbatim. Data were coded inductively and deductively using the software program MAXQDA (VERBI Software, Berlin, Germany, MAXQDA Plus 2018 Version 18.2.0). The analysis of data was guided by the principles of qualitative thematic analysis proposed by Braun and Clarke [38], using a codebook approach [39]. Taking an iterative approach, the first author read and open coded the transcripts. After each transcript, the coding was checked by and discussed with the second author in order to develop an initial code tree. The code tree was then used to (deductively) assign codes to relevant passages of previous and further interview transcripts, allowing for additional (inductive) codes when new topics emerged. No new topics emerged after coding focus group number six. After coding was completed, codes were further refined and reduced in number by revising them and grouping them together into categories. To identify potential themes, all coded data were examined by interpreting recurring items, topics and support needs. To inform the themes, we also searched for similarities and differences between the mentioned support needs by participants and the topics they addressed [40]. Going back and forth between the data, the content of the (sub)themes were refined by the first author and the coherence and distinctness of the themes were discussed between the first and second authors until consensus was reached. For triangulation purposes, we checked and enriched the identified themes against other data sources that were collected during the study. This also included a session with the advisory board, in which they were asked to reflect on and add to the identified themes.

## 3. Results

### 3.1. Participant Characteristics

Five female and two male working caregivers participated in the advisory board of the study. Additionally, a total of 25 working caregivers participated in the FGDs, of whom 22 were female and 3 male (Table 2). Group sizes varied from three to five participants. The mean age of the FGD participants was 53.7 (±1.6), ranging from 28 to 68 years. Caregivers could list the sociodemographic information of only one care recipient, but 15 caregivers indicated that they provided care for multiple care recipients simultaneously (for example, for their partner under 65 years old and a parent). The mean age of the care recipient whose age was reported by the caregiver was 82.3 (±1.9), ranging from 57 to 99 years. The average number of hours per week spent on caregiving was 14.6 (±3.2) (range 1–75), and they worked an average of 28 (±2.8) hours per week. The relationship between the caregiver and older adult was mostly child–parent (22 out of 25 caregivers), while two caregivers took care of a partner and one of a grandparent. The caregiving tasks that were listed were diverse, ranging from household chores, transport and social visits to medical care and care coordination.

### 3.2. Support Needs of Working Caregivers

**Figure 1:**
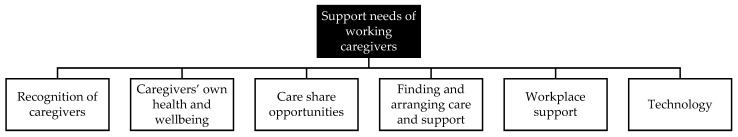
An overview of themes related to working caregivers’ needs.

Working caregivers expressed several needs for which they could use support or where support could be improved. Six themes, covering the caregivers’ support needs, were identified from the data: (1) Recognition of caregivers, including the challenges they face; (2) Attention for caregivers’ health, wellbeing and ability to cope; (3) Opportunities to share care responsibilities; (4) Help with finding and arranging care and support; (5) Understanding and support from the work environment; (6) Technological support tailored to the needs and capacities of caregivers and older adults. An overview of themes mentioned by the participants is presented in Figure 1. To address their needs, caregivers suggested several support options in different settings (i.e., home and social environment, care and support environment, work environment and societal/policy environment; Table 3.

Theme 1: Recognition of Caregivers, including the Challenges They Face

The caregivers in this study advocated for more awareness and recognition of caregivers in society. They felt that people could be better informed on matters such as when one can be considered a caregiver, what it means to be a caregiver and the daily challenges they face, including difficulties in combining work and care. This would be helpful to them in two ways. Firstly, they believed it could help caregivers to better recognize themselves as a caregiver, as several participants mentioned that they just enrolled in their caregiving roles without being conscious of it and the consequences.

“*Again, it’s about everyone being familiar with it. You know, it starts with awareness. If you don’t actually realize that you are a caregiver and whatever that entails, you know, you just do it, you take care of your loved ones anyway. And the situation presents itself and before you know it you are in the middle of it and you are so caught up in it, that you get sucked into that flow and only later on you find out, wow, that flow is fierce; what am I doing?”* .

(Part. 2 FGD 4, female)

Several caregivers mentioned being unaware of the possibilities to receive support and discussed how not considering yourself a caregiver could hinder the use of support services available for caregivers.

“*It was my son who said: ‘Now you have to slam on the brakes, because of course, Mom needs that care, but you don’t have to do it alone. You can get support for that.’ And it’s not like I was afraid to ask for help there, but it had never occurred to me. Never.*”.

(Part. 2 FGD 5, male)

Secondly, more recognition of caregiving and its challenges would help caregivers feel more visible and appreciated in their role. Some caregivers would like to be more acknowledged by health and social care providers, while others mentioned supervisors at work and colleagues, or the older care recipient themselves. They suggested that societal recognition could be spread through informational campaigns (e.g., posters, flyers and commercials) by the government or by employers.

“*But you know, I never get any recognition. It’s all taken for granted. So if there is just a lot more attention for this from society, then I also think it is something that… In the end I think that every person wants to be recognized in that way too, and that you will be able to carry on longer in that case*.”.

(Part. 2 FGD 4, female)

Although caregivers saw more awareness for caregivers as a good general improvement, they emphasized that better societal recognition should also entail improving actual support structures and caregiver rights in society. They noted that, although there is much societal demand for informal care, they did not always feel sufficiently supported in their roles.

“*We have a societal issue here in the sense that we think that informal care is very important. So then it should be organized, right? Don’t go around saying, “Just be a caregiver, and how? Just go and figure it out for yourselves.” These people all have to reinvent the wheel, so why not make it a law, do something with it in the collective labor agreements so that it is protected, that you have rights as an informal caregiver. That it is a right, not only a duty and something you want, but also a right that you can and are allowed to do*.”.

(Part. 2, FGD 5, male)

Theme 2: Attention for Caregivers’ Health, Wellbeing and Ability to Cope

Some caregivers found it difficult to balance work and care with their personal lives. They seemed to prioritize their work, family and caregiving tasks above their own wellbeing: *“Keeping all balls in the air all the time… Whatever happens to be the greatest need at the moment, that’s what will take up most of your time. That’s how it came across to me. And you don’t spend the time on yourself, in any case.”* (Part. 3, FGD 6, female). Caregivers mentioned several personal adverse effects of having to juggle care and work demands, such as health problems, fatigue and limited room for relaxation and exercise. They indicated to sometimes experience feelings of anxiety and guilt if they were not around to give care, and were then unable to enjoy their idle time. Moreover, some caregivers experienced negative spillover effects into their family lives, reporting marital problems, neglecting household chores and being unable to spend enough time with one’s children or partner. Although they emphasized that they saw caregiving as a labor of love from which they often derived fulfillment, it was sometimes hard to truly disconnect and recuperate from their caregiving responsibilities.

“*Well, you call twice a day, right? And during our holiday, we went to my mother-in-law, and I went to see my mother. And it just keeps you busy. You’re never completely away from it all*.”.

(Part. 3, FGD 5, female)

Caregivers expressed several needs, centered around helping them to take better care of their own mental health and wellbeing. An often-mentioned need was having a ‘sympathetic ear’ to share their situations, questions and concerns. This could for example be someone in the caregiver’s family or social network, other caregivers or a buddy at work. Talking to other caregivers could be mutually beneficial as they could exchange advice and tips based on shared experiences. Others preferred support from a professional who is at a distance from the caregiving situation, such as a psychologist or coach. Such professionals could help caregivers to deal with different challenges, such as setting boundaries in their role as a caregiver and delegating tasks. Caregivers described caregiving as a ‘sliding scale’ in which they take on more and more tasks without realizing. This implies a risk of being overburdened when they recognized too late that they needed support.

“*Yes, [what I need] is only one thing and that is a sympathetic ear, someone who wants to listen to my side of the story only. It could be on the phone, or in a live chat. I wouldn’t mind if that’s planned, if I say, ‘I just need a little chat now,’ that we schedule that. I’m thinking of a coach or something along those lines, who is good at asking questions, is able to confront and also helps to maybe think about possible solutions [...] Someone who really notices: now you are of no use to anyone, now you have to take a break*.”.

(Part. 5, FGD 2, female)

“*[I would like to] learn how to take good care of myself. Making sure that you don’t get completely exhausted. It would be good if this comes into the picture not just when you get stuck or are in danger of complete exhaustion or are getting close to a burnout, but much earlier. […] So that is also something that can be discussed when it comes to supporting the caregiver*.”.

(Part. 4, FGD 6, female)

Theme 3: Opportunities to Share Care Responsibilities

To alleviate care burden and prevent overload, caregivers also mentioned having a need for more opportunities to share and delegate their care responsibilities. They expressed a need for both incidental support (e.g., for a vacation or a day off, to take over some social visits or to go with the care recipient to doctors’ appointments) and structural support (e.g., respite care, ongoing home care or adult day services). Some caregivers preferred more assistance with (medical) care tasks, while others were in need of more support with day-to-day tasks, such as keeping the older adult company and helping them live comfortably at home. To be able to recharge, the participating caregivers preferred sharing the care with someone that they themselves and the care recipient are familiar with and trust: *“That you just know: there is a trusted person who will now take over from me and I can let things go for a bit and, because of that, pick things up again with good energy, as it were.”* (Part. 3, FGD 1, female). Caregivers, therefore, stressed the importance of supporting caregivers early on in helping them build a preventive network to fall back on and share caregiving tasks.

“*Networking before informal care is needed; a social basis is important. There is not enough attention being paid to that at the moment. Everything is focused on care and not on knowing each other, and sharing things with each other and staying out of care. [That’s why we need to] build a preventive network so that if something happens, then you have a network that is a logical interlocutor and considers it normal to offer support for a while*.”.

(Advisory board member, female)

Caregivers mentioned several barriers that prevented them from making use of support in sharing care, such as having a small personal network to rely on or the older adult’s refusal of (extra) care from strangers. They also experienced financial barriers to make use of private care initiatives that provide care and company at home. Lastly, caregivers mentioned knowledge barriers, such as a lack of awareness of private and municipal support services to share care.

Theme 4: Help with Finding and Arranging Care and Support

Caregivers felt a strong responsibility for their loved one’s health and wellbeing. Apart from their personal caregiving tasks, they often dealt with practical struggles and regulatory matters while arranging the older adult’s care and support. These coordinating matters were often experienced as time consuming and seriously burdensome: *“The maze of everything you have to figure out for yourself drives me completely nuts. […] I am already in a situation where I can no longer even think clearly and logically.”* (Part. 6, FGD 3, female). According to the caregivers, a major problem was posed by the ‘jungle’ of health and social care services and providers which they had to navigate, each with their own rules and regulations. They found it important that the accessibility of the care system improved for both caregivers and the older care recipients, in particular for those who were new to the system or experienced language barriers, for example due to a migration background.

“*As an informal caregiver, you’re supposed to do that [arranging care] yourself, but you can’t. Certainly not if you don’t speak the language and certainly not if you’re new to it and don’t even know where to start. So then it really depends very much on: are you lucky enough to meet the right people who will take you by the hand and guide you and help you on your way? And if not, it is a real struggle to organize the right care and support at all before you get to… look after yourself as a caregiver. […] And the moment the care institutions are more prepared for that group of older migrants, this can ease the burden on caregivers more quickly*.”.

(Part. 4, FGD 6, female)

“*You don’t know where to start, you want to help, but there are a hundred thousand regulations, a hundred thousand service counters, and how does it all work?*”.

(Part. 3, FGD 6, female)

Other recurring issues mentioned by the caregivers were communication problems with and between different health and social care providers, it only being possible to reach care providers and plan appointments during working hours and privacy regulations that hampered the arrangement of care in the name of the older adult. Caregivers, therefore, voiced a strong need for assistance with the managerial challenges related to these processes and procedures. Likewise, caregivers also experienced difficulties with finding and arranging support for themselves.

To better help caregivers in the processes of finding and arranging care and support —both for themselves and the older care recipient—participants needed a clear and easy to find overview of all available support services. Such an overview should include where and how to apply for the support, such as an (independent) platform, a website, a toolkit, a step-by-step plan, a central telephone number or an information desk at the municipality. *“Finding information in an accessible way […]. I find it very difficult to find decent information and how it all works and what you have to do and what you have to consider.”* (Part. 3, FGD 5, female). In addition, the caregivers expressed the need for a central point of contact, such as a case manager or municipality employee, who can offer practical advice on the appropriate support for one’s situation and proactively assist in the arrangement of care.

“*I just wish there was a central point, where someone is assigned to you. Someone who actually helps you to sort out how you can best get things organized. And who maybe also takes some steps in that sense. Because I think now it’s really… it’s enough to make you weep*.”.

(Part. 6, FGD 3, female)

“*It would be so good if you had one service counter at the beginning, at the starting point. Or one person with whom you can get things started and who puts you on the right track, what the options are. So the starting point should be clearer and easier to find*.”.

(Part. 3, FGD 6, female)

A particular concern of caregivers regarding the organization of care was not being sufficiently prepared to deal with potential future changes in the health and care of their loved one. For example, they worried about how their loved one could continue to live comfortably and independently at home, or how to best arrange the transition to a care home. They, therefore, preferred that a contact person would also help them anticipate these future challenges so that they can act and prepare in a timely manner:

“*What I can expect and whether they think that my mother can continue to live independently. And if not, should I be taking steps now, should I go and start inquiries at the nursing home… Yes, all these things actually. What I should be considering and what I can work towards, as it were*.”.

(Part. 3, FGD 4, female)

Theme 5: Understanding and Support from the Work Environment

According to the caregivers, having to combine paid work and care should be seen as a shared issue between employer and employee, and not just as a personal problem. Caregivers, therefore, found that employers should offer support to their staff who deal with caregiving tasks. Needs that should be addressed by the workplace/employer were: (1) more social support from employers, supervisors and colleagues, (2) expanded and tailored solutions to help caregivers combine paid work and care and (3) increased knowledge of the rights and support options for caregivers among both employers and employees.

Social support at work in terms of understanding of the employer, supervisor and colleagues was considered important. The caregivers indicated that employer understanding would make them feel supported and less guilty about their possible suboptimal work performance. This was supposed to imply an open and safe culture in the workplace where such issues can be openly discussed. However, several caregivers actually found it difficult to discuss their situation and truly convey what kinds of challenges they encounter as a working caregiver.

“*And I myself was thinking more about, well, understanding from your employer and your colleagues. You can try to explain… It’s sometimes very difficult to explain what’s happening to you, so that they can also better understand that some days you can do less—less of your work. That you’re there, but you’re not actually there*.”.

(Part. 4, FGD 2, female)

“*Well, my most important need is really understanding. You know, understanding from your employer. When you are really busy with care, no matter for whom, that there is understanding and openness and ... safety, especially the safety of being able to share and talk about it. That sharing is encouraged, that you can also communicate and name it*.”.

(Part. 2, FGD 6, female)

While some caregivers reported positive experiences with the understanding they received from their employer (*“And when something happens unexpectedly—they never mind that, when I tell them, they always say: ‘That’s a priority.’”* (Part. 3, FGD 4, female)), others encountered a less empathetic attitude: *“And I always indicate this [the care tasks] at the start of work, also to my employer. And I’ve just noticed that there is very little room for that, very little understanding. You know if you are an engine driver, then you have to work. [...] And I actually experienced that my care tasks at home were perceived as very difficult [by my employer].”* (Part. 2, FGD 5, male).

Apart from social support from their employer, caregivers needed tailored solutions to be offered to combine paid work and care. For instance, some preferred more flexible and continuous opportunities to take care leave—preferably without a loss of income. Long-term caregivers, in particular, found current care leave options not always suitable for their situation, which meant that they did not use them or used vacation days instead. Furthermore, caregivers required flexible working hours and possibilities to work outside of the office—which had become easier in some cases during the COVID-19 pandemic—but also mentioned that they used this flexibility to catch up on work during evening/night hours, which left little time for themselves or their families. It was, therefore, also considered important that employers support them by (temporarily) diminishing their task load or reassigning duties to someone else.

Likewise, some of the self-employed caregivers emphasized that they also had a need for financial support structures and care leave: *“There is nothing to fall back on, to support you at that time, to make things a bit easier. And that was pretty scary, that I thought: I can’t afford a burnout. There is no sickness benefit if that happens, there is just nothing I can turn to.”* (Part. 3, FGD 6, female).

Finally, some caregivers mentioned that they were unaware of their rights and the existing organizational policies for caregivers, and would like to be better informed about their options. *“To be honest, as a caregiver, I don’t even know what kind of laws about that there are. You know, your employer’s regulations and laws. I don’t know them. It would be great if something could be done about that too.”* (Part. 2, FGD 6, female). Others also indicated employers themselves were ill-informed about available workplace support: *“The employer should also make a plan for informal caregivers. It’s not as if you are the first to ask the question: I need care leave. They still had to think about it.”* (Part. 2, FGD 3, female).

To address all these needs, caregivers stressed the crucial role of (direct) supervisors. The participants who were satisfied with their options from the employer to combine work and care often mentioned that support, in the form of feeling heard and understood by their supervisor, played a role in their positive experience. Conversely, negative experiences with supervisors could contribute to feelings of burden and dissatisfaction in being able to combine paid work and care.

“*Over the years, I have experienced different reorganizations and had different managers. And usually I could count on the understanding and cooperation and flexibility of the manager. But I have also had a manager who had no sympathy at all. Who thought that those kinds of privileges should be stopped*.”.

(Part. 4, FGD 6, female)

Theme 6: Technological Support Tailored to the Needs and Capacities of Caregivers and Older Adults

The majority of the caregivers mentioned that they or the older adult they were taking care of (had) used some form of technological support, such as technology to stay in touch with the older adult (e.g., video calling), to ensure the older adult’s safety (e.g., hip airbag, portable alarm button, GPS tracker) or to communicate with other caregivers or care professionals. However, experiences with technology were mixed.

“*Yes, we had one of those sensors, like a chain, you know, that my mother could press if something happened in the house. It was linked to our phone numbers, but she just didn’t want that. She kept throwing it out and it was a waste of money, because you had to pay for it every month, for the switchboard. But for her, it had no added value*.”

(Part. 4, FGD 4, male)

In general, caregivers were positive about the use of technology in caregiving when it added value for both the caregiver and older adult. According to caregivers, technology could be beneficial when it relieves them from worrying about the older adult’s safety, for example through the use of alarm buttons or updates about the older adult’s day in an online ‘family environment.’ To facilitate the use of technology by the older adult, some prerequisites were mentioned, including an open, positive attitude towards the use of technology and technology that was user-friendly and tailored to their cognitive and physical capabilities.

“*He just has those fingers with rheumatism, so it’s not always that easy. So if he has to do something on his tablet, it often goes like, you have to enter a code and then three of the four numbers are entered, but not the fourth and then it goes wrong again. And that can be a problem for him. So the tools are often not really geared to being used by the elderly*.”.

(Part. 2, FGD 2, female)

Still, the caregivers mentioned that it takes time and effort to become familiar with the use of technology and its benefits: “*Of course, we think very quickly and very easily, but that is not the case in their lives, so it takes a lot of patience and time and explanation from the caregiver, to get things done. And once it’s there, then after a day he says, ‘Oh, I wish I had done it much earlier*.’” (Part. 3, FGD 2, female). They, therefore, recommended that caregivers should start the introduction of technology as soon as possible, so that the older adult can get familiar with it and experience less barriers in its use. To help in the actual application of technology, assistance or teaching through digital coaches or through technology workshops were suggested.

Finally, caregivers also expressed a need for more knowledge on what kind of technology is available to help older adults to live longer at home: *“We talked earlier about looking ahead and future scenarios. And we happened to come up with this idea [of a stair lift], but I’m also sure there are all kinds of things I never thought of that I’m going to come up against later on. And, yeah, it would be nice to just have a better idea of that.”* (Part. 2, FGD 2, female).

## 4. Discussion

The aim of this study was to explore the support needs of working informal caregivers taking care of an older adult. The needs of the caregivers could be clustered around the following six themes: (1) Recognition of caregivers, including the challenges they face; (2) Attention for caregivers’ health, wellbeing and ability to cope; (3) Opportunities to share care responsibilities; (4) Help with finding and arranging care and support; (5) Understanding and support from the work environment; (6) Technological support tailored to the needs and capacities of caregivers and older adults. The results show that caregivers need support to balance caregiving with other aspects in their lives, including paid work, home life and their personal health and wellbeing. Support is needed across multiple settings and domains of life (i.e., home and social environment, care and support environment, workplace and societal/policy environment (see also Table 3), and should be tailored to the caregivers’ and care recipients’ contexts. This implies involvement of several actors in the provision of support (e.g., health and social care professionals, actors within the work environment, local policy officers etc.), including the caregivers themselves.

### 4.1. Caregivers’ Personal Needs

Regarding caregivers’ personal needs, findings of this study underline that, to help them to maintain their wellbeing while combining work and care, caregivers required more formal (e.g., care professionals) and informal support (e.g., friends, family or other caregivers) than they currently receive. An important starting point is to help caregivers realize that they are indeed caregivers and that support options are available to them. This implies that persons around the caregiver and care recipient, such as the social environment, health and social care professionals and employers, should acknowledge caregivers for their roles, and pay more attention to their needs [28]. Furthermore, as was also found in other studies, caregivers needed support aimed at increasing their capacity to cope with the caregiving demands and situation. This included helping them recognize their own needs and limits, and adapt to future changes in the health and care of their loved ones [30,41,42]. Our findings align with a growing body of literature that focuses on increasing caregiver resilience, i.e., the ability to adapt to changes and challenges in their own and the care recipients’ lives, as a potentially effective way to support caregivers [34,43,44,45]. The findings in this study further contribute to this knowledge by providing several ways in which caregivers could be empowered to improve their resilience, e.g., by providing coaching, professional support (e.g., help in anticipating future steps), caregiver support groups, training and information to caregivers. This information can be used by policymakers, local authorities as well as health and care providers to develop and implement suitable support services aimed at working caregivers.

### 4.2. Needs Within the Care and Support Context

Besides support specifically targeted at themselves, caregivers also needed help within the care and support context, related to the management and coordination of care of the older adult. Caregivers felt highly responsible for ensuring the continuity and quality of care for their loved ones, and they spent significant amounts of time and energy on these managerial tasks. Our results indicate that improving services for the older care recipient, as well as the procedures and accessibility of care and support services, could contribute to reducing working caregivers’ burden. As was also found in other studies [28,42], caregivers could benefit in particular from clear information about all available support options and procedures (e.g., for caregivers, care recipients, technological support etc.), and could also benefit from a fixed point of contact that can help them access a range of public and private services.

In the Netherlands, different parties are responsible for the care and support of older adults and caregivers, which means that caregivers can have different ‘entry points’ when seeking help, such as their GP, community health and social care organizations, voluntary care organizations or local authorities. Additionally, some support options, such as municipal client support services (i.e., a trained professional or volunteer that helps vulnerable citizens to find and arrange care and support) seemed largely unknown or hardly used. To better assist caregivers and address their multiple needs, each organization may do well to appoint professionals who are familiar with or well-informed about available care and support options—and can also refer caregivers to support by other organizations or in other sectors. This implies an active knowledge exchange and increased collaboration between different parties. We also recommend health insurance providers to explore options to provide a fixed point of contact or ‘case manager’ to caregivers who need help figuring out the (next steps in the) arrangement of care. In the Netherlands, such a service is usually only provided when the older adult is diagnosed with dementia. However, our results suggest that caregivers in other care situations may also benefit from such support. Earlier studies have demonstrated the added value of case management in cases of dementia, chronic illness and frail older adults with complex care needs [46,47,48].

Some support services may serve both caregivers and older adults (e.g., respite care, adult day care). In these cases, it is important to realize that preferences regarding these services may differ between caregivers and older adults. Our study, for instance, shows that caregivers sometimes found it difficult to arrange care from third-party persons, or to introduce technology that the older adult had to use, such as alarm buttons connected to the caregiver. While caregivers may perceive care sharing and technology as a way to lower burden, the older care recipient may see this support as unnecessary or as being coddled, or they might even find it unpleasant to receive care away from home or from strangers [49,50]. More research is needed into how the (in)congruent preferences of caregivers and care recipients actually impact the use of support services, and how support can be improved to better match the preferences of both.

### 4.3. Needs within the Work Environment

Regarding the caregivers’ needs in the work environment, our findings show that fostering mutual ‘understanding’ is an important first step in order for caregivers to feel seen, heard and supported in the workplace. This requires effort from both the caregiver and employer. Even though caregivers perceived the work–care combination as a shared issue between the employer and employee, they sometimes found it difficult to share and explain the daily challenges they face. Other studies have found that barriers that keep caregivers from sharing their situation are work cultures where you need to be perceived as always ‘coping,’ and fear of being seen as a ‘weak’ employee and missing out on job opportunities [51,52]. Working caregivers could benefit from support that focuses on empowering them to discuss their caregiving situation and formulating their needs towards their supervisors and colleagues. More research is needed into how caregivers could be better facilitated herein, and the accompanying role of supervisors and co-workers. On the employer’s side, our findings underline the importance of co-workers, and in particular supervisors, offering social support. Employees who feel that their wellbeing is valued and supported by people from their workplace have been shown to experience reduced work–family conflict and caregiving strain [53]. Additionally, employers should provide more information on the rights and policies for caregiving employees, and should also offer customized support that fits their individual needs (e.g., care leave options, flexible work arrangements and adaptations in workload or duties). Although effects of different caregiver workplace interventions have not yet been extensively explored [54], flexible work arrangements are often mentioned as a way to relieve burden in caregiving employees. This was also found by van Echtelt et al. (2016), who demonstrated that flexible work arrangements could assist caregivers in keeping up with both work and care demands, but in doing so could also increase burden and combination pressure [55]. In our study, caregivers who could work flexibly sometimes mentioned working late into the night to keep up with work demands. We, therefore, recommend employers to also be open to adapting working caregivers’ task load when the situation calls for it. Further investigation is needed into how to better stimulate and facilitate an open discussion taking into account the needs, possibilities and preferences of both the employer and employee with caregiving tasks.

### 4.4. Timing of Support

Finally, our findings underline that caregivers may actually need support in different stages of the caregiving process, and not only when the care load is heavy. In fact, it appeared that the caregivers in this study experienced a particular support gap in earlier stages of caregiving, when they wanted to help the older adult to continue to live comfortably at home, and prepare for future steps and changes in the caregiving situation. Taking an upstream approach by providing support for early-stage caregivers could contribute to preventing overload and high burden later on [56].

### 4.5. Methodological Considerations

In this study, we took working caregivers’ own experiences as the starting point to comprehensively explore their support needs and the challenges they encounter, which is a strength of this study. Since caregiving occurs within a multi-faceted context, the identification of support needs was not restricted to a specific type of support or setting (such as the work environment). To ensure a broad representation of views, we made efforts to include working caregivers with a variety of caregiving and employment situations. Additionally, data were triangulated and enriched with the experiences of working caregivers in our advisory board.

Even so, however, some caregivers were underrepresented in this study, including male caregivers, caregivers with a low education and those with a migration background, which is a limitation of this study. These groups may have specific support needs and experience particular challenges that may not all have sufficiently been captured in this study. For example, male caregivers may require support for the specific challenges they encounter, as was indicated by one male caregiver, who preferred a support group aimed at male caregivers and their experiences. Additionally, many caregivers in this study experienced difficulties navigating care and support systems, but mainly caregivers with higher education levels were included. Lower-educated caregivers arguably may experience additional challenges in managing the care of their loved ones, and may also require targeted solutions improving accessibility and information supply. The inclusion of more male participants and caregivers with lower education may, therefore, have yielded additional information regarding support needs and themes. More research is necessary to investigate how and in which cases support needs of these groups may differ from other working caregivers, and how support may be adapted to meet the needs of different targeted groups.

Lastly, due to COVID-19, we organized the FGDs online instead of in person. Online interaction may be experienced as intensive and more difficult due to reduced visibility of context cues (such as body language and other non-verbal behavior), which can hamper active participation and effective moderation [35]. We made efforts to mitigate this by including different discussion techniques, which added variation to the discussion and ensured that each participant had equal opportunity to contribute.

## 5. Conclusions

Based on this study, we conclude that working caregivers have support needs in multiple settings and domains of their lives. To better support working caregivers, efforts in different settings and taken by several actors are needed, including the caregivers themselves. This makes the provision of adequate support for working caregivers a multi-faceted effort. In general, caregivers felt like their roles and challenges should be better recognized and valued in society, which implies that people around the caregiver need to pay more attention to their needs. On a personal level, caregivers need support in increasing their capacity to maintain their own (mental) health and wellbeing while giving care. In the home and social environments, caregivers require digital assistance, tailored technological support and opportunities to share their care load and concerns with others. Within the care and support context, caregivers need help finding and arranging care and support for both themselves and their older loved ones. Finally, in the workplace, caregivers find it important that the environment shows understanding for their situations and challenges, while also offering support to better combine work and care. Taken together, the results of this study can inform research, policy and practice to improve and implement support aimed at working caregivers of older adults, in different settings.

## Figures and Tables

**Table 1:** Discussion guide for the online focus groups.

**Question 1**	What are your experiences and what challenges do you encounter, while combining work, life and care?
**Question 2**	What are your support needs as a working caregiver, and how can your needs be met?
**Question 3**	How can technology or ‘e-health’ be used to support you as a working caregiver?
**Question 4**	What constitutes good caregiver support according to you?
**Question 5**	What can be done to help working caregivers who need support, to find their way to the right support in a timely manner?

**Table 2:** Characteristics of caregivers participating in the focus groups.

	Total (*n* = 25)
**Gender**	
Female	22
Male	3
**Hours of Caregiving Per Week**	
1–10 h	14
11–20 h	6
21–30 h	3
>30 h	2
**Educational Level**	
Unknown ^1^	1
Low educational level	0
Middle educational level	4
High educational level	20
**Hours of Paid Work Per Week**	
<20 h ^2^	5
20–30 h	9
31–40 h	9
>40 h	2
**Employment**	
Not currently working ^2^	3
Salaried employment	14
Self-employed	7
Self-employed and in salaried employment	1
**I am Satisfied with How I am Able to Combine Work and Care**	
Strongly agree	3
Agree	9
Neither agree nor disagree	7
Disagree	4
Strongly disagree	2

^1^ One participant had finished a high school education, but did not specify which level. ^2^ (Non-pilot) participants were included in this study if they had provided care in the previous five years, while working at least 20 h per week. However, at the time of completion of the questionnaire, several caregivers had reduced their working hours or stopped working altogether.

**Table 3:** Support options in different settings mentioned by caregivers participating in the focus groups.

Home and Social Environment:
Technological support for the older care recipient (e.g., portable alarm button and GPS, sensors in and around the house, ‘walking airbag,’ tablet) or caregiver (planning and communication apps). A listening ear for the caregiver from the social environment. A helping hand from the social environment (e.g., a check-up by neighbors of older adult, sharing care tasks).
Care and Support Environment:
*Information:*
An easy to find overview of all available care and support services for caregivers and older adults.
*Services:*
A fixed point of contact who can help to find and arrange the appropriate care and support. ‘Digital’ coaches for continuous assistance with (new) technology. Training and courses for caregivers developing personal skills (e.g., mindfulness, adapting to changing care situations, setting boundaries in caregiving tasks). Caregiver support groups or ‘walk-in hours’ for caregivers in the community. Coaching/advice by professional psychologists or coaches, focused on the caregiver’s ability to cope. More options for respite care close to home or delivered at home. An index of back-up caregivers (professionals, volunteers) that you can easily call on. Assistance in forming a care network around the older care recipient early on. Household help for the caregiver. A service where someone else arranges replacement care for the caregiver.
*Process improvements:*
Possibilities for (planning) appointments for care and support outside office hours. Possibilities for online appointments for care and support. Improved communication between caregivers and health and social care providers. Less administrative burden in applying for care and support. Improved privacy regulations, making it easier to arrange support in the name of the older care recipient.
Work Environment:
A point of contact to discuss work–life balance with (e.g., with supervisor, a confidential counselor, occupational physician or a work and care ‘attention officer’). Training supervisors in initiating a conversation with employees about work–care balance. Employer campaigns spreading awareness about employees with care responsibilities. Flexible and continuous opportunities to take care leave without a loss of income. Flexible work arrangements (working from home and outside of office hours). Adaptions in work organization and task load of the caregiver. Information and training for supervisors and employees on the rights and support options for caregivers.
Societal/Policy Environment:
Informational campaigns spreading awareness of the concept of caregiving and what caregiving entails. Establishing rights for caregivers in collective labor agreements across all sectors, as well as rights (e.g., care leave and financial support) for self-employed caregivers. Compensation for psychological and lifestyle support for caregivers through health care insurance.

## Data Availability

The data presented in this study are not publicly available due to privacy regulations. For more information about the interviews and transcripts, please contact the corresponding author.
